# Mycobacterial Preauricular Sinus Abscess: A Case Series

**DOI:** 10.7759/cureus.44287

**Published:** 2023-08-28

**Authors:** Eric Wang Yuan Li, Siti Halimahtun Sahab, Noorizan Yahya, Mohamad Khir Abdullah, Noor Dina Hashim

**Affiliations:** 1 Otorhinolaryngology - Head and Neck Surgery, Universiti Kebangsaan Malaysia Medical Centre, Kuala Lumpur, MYS; 2 Otorhinolaryngology - Head and Neck Surgery, Hospital Pakar Sultanah Fatimah, Johor, MYS

**Keywords:** tuberculosis, preauricular sinus, preauricular sinus abscess, ear pits, mycobacterium tuberculosis, extrapulmonary tuberculosis

## Abstract

Preauricular sinus is a common congenital external ear anomaly. It occurs due to the incomplete fusion of hillocks of His of the first and second branchial arches. Tuberculosis (TB) is endemic in Malaysia, which imposes a major public health problem. It is caused by *Mycobacterium tuberculosis*, which causes chronic, recurrent diseases and poor healing of a wound. Pulmonary TB is the most common form of infection, some manifesting as extrapulmonary TB. We share our experience in managing a series of three patients with recurrent tuberculous preauricular sinus abscesses in different age groups. Testing for acid-fast bacilli is highly advocated in recurrent cases and in extensive infection of preauricular sinuses despite the absence of systemic or pulmonary symptoms. Treatment with anti-tuberculous drugs is commenced, followed by an elective sinus excision once the patient is free from infection to prevent recurrence.

## Introduction

Preauricular sinus is a common external ear congenital abnormality [1]. Patients with preauricular sinus may not present with any symptoms [2]. Some patients present with discharge, pain, and redness from the preauricular sinus, which can get infected causing preauricular sinus abscess [3]. They become infected commonly with staphylococcal species and less commonly with *Streptococcus*, *Proteus*, and *Peptococcus* species [1,4]. Preauricular sinus abscess infected with *Mycobacterium tuberculosis* is very rare, with only one case report found in a review of the literature in Malaysia and one in Thailand [5]. In a chronic recurrent infection of the preauricular sinus, systemic antibiotics should be administered, followed by incision and drainage if an abscess is suspected, and pus should be sent for culture and sensitivity and acid-fast bacilli (AFB) [5]. Tuberculous preauricular sinus abscess should be considered if it does not respond to conventional antibiotics or recurs after surgical drainage [5].

## Case presentation

Case 1

**Figure 1:**
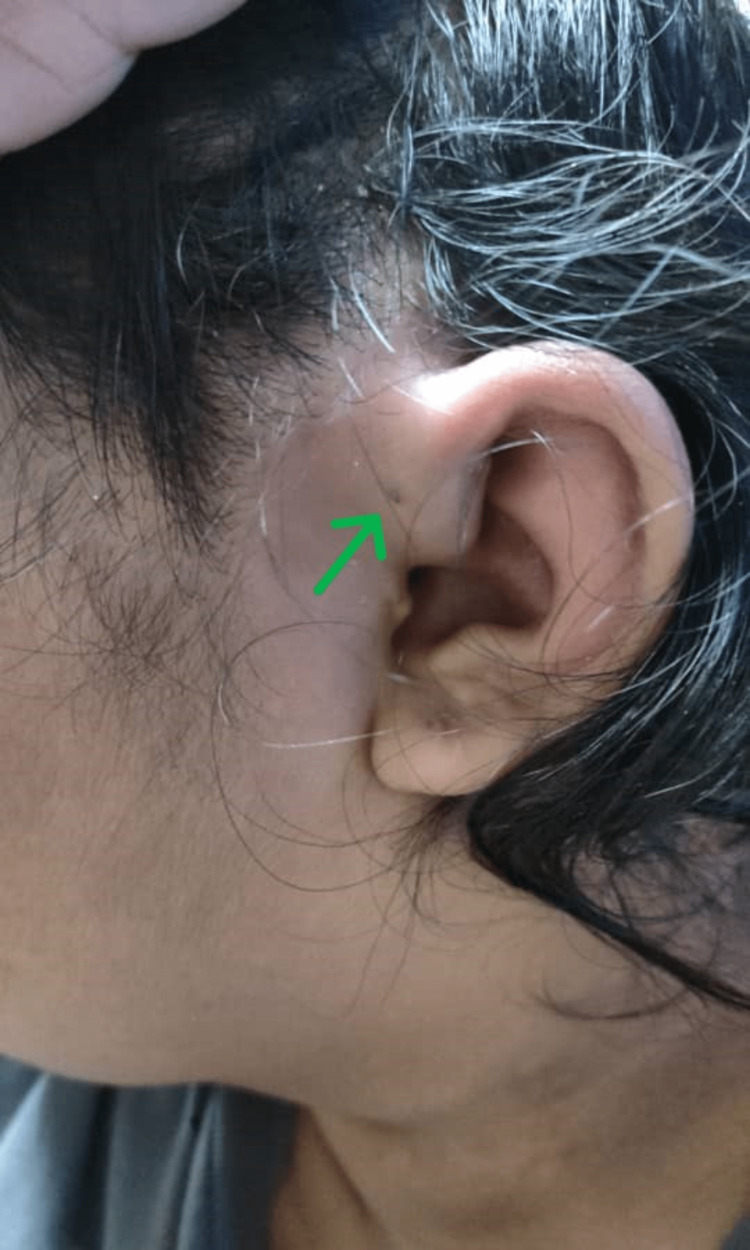
The green arrow shows left preauricular swelling with a preauricular sinus opening. No active discharge was observed.

**Figure 2:**
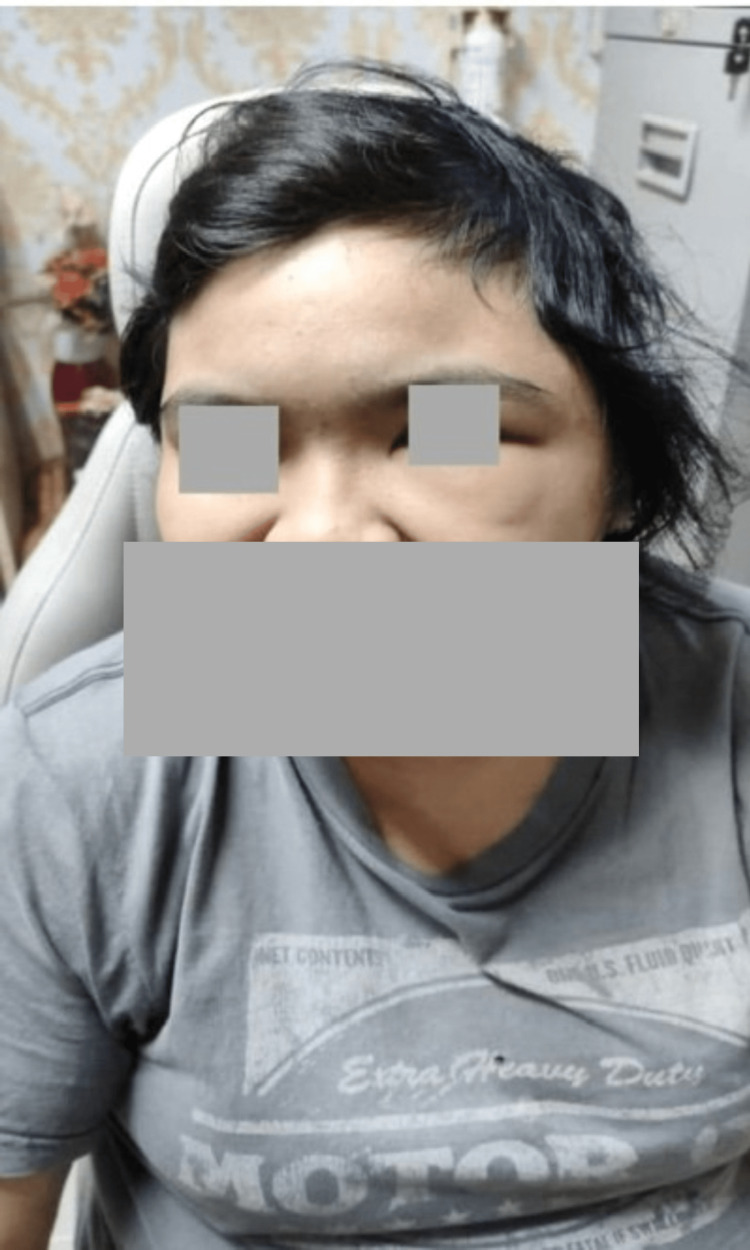
Left preseptal cellulitis.

A 20-year-old lady with no known medical history of interest presented with swelling around the left preauricular region for five days associated with left eye swelling. She denied having ear discharge, fever, or nasal symptoms. On examination, there was a tender, fluctuant, left preauricular swelling measuring 2 x 2 cm. There was no active discharge at the sinus opening (Figure 1). There was an extensive left periorbital edema with no ophthalmoplegia observed (Figure 2).

**Figure 3:**
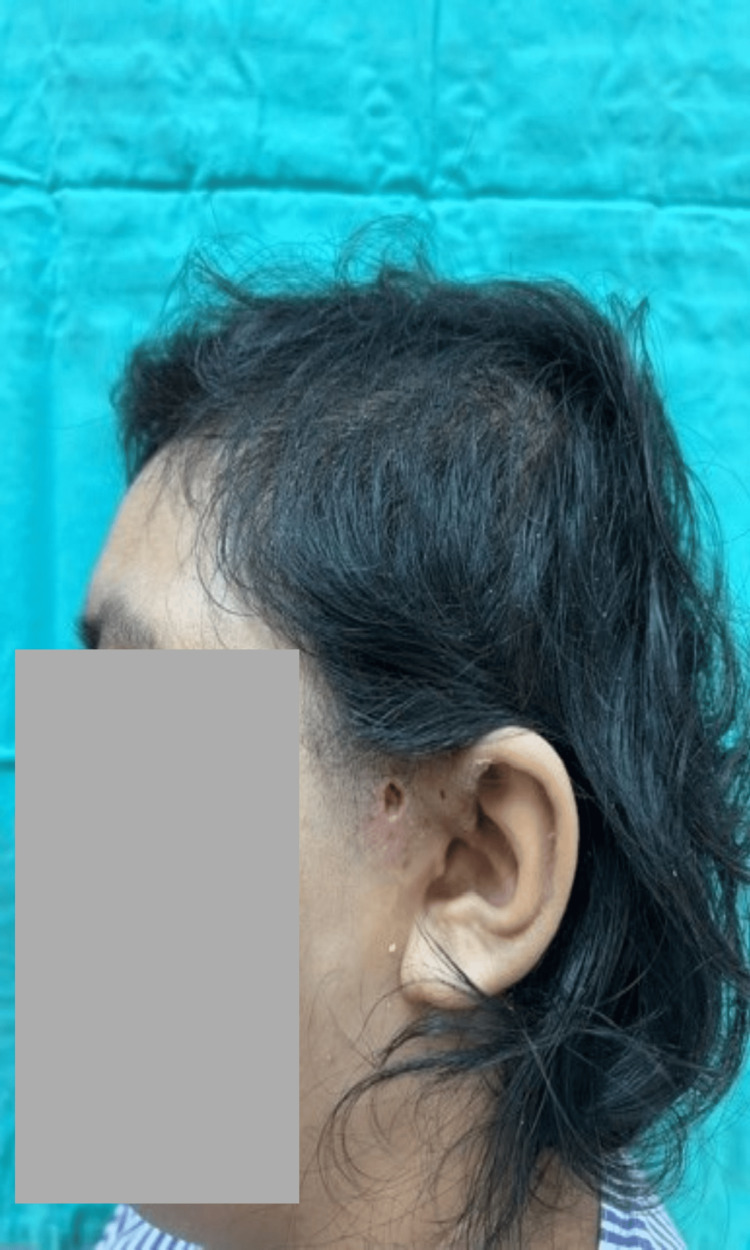
Left preauricular sinus abscess showing resolution of swelling and preseptal cellulitis.

A diagnosis of left preauricular sinus abscess with preseptal cellulitis (Chandler class I) was made. She was subsequently admitted and started on intravenous amoxicillin and clavulanate and scheduled for drainage under local anesthesia. In view of the extension of infection to the periorbital region, a sample was sent for culture and sensitivity as well as for AFB. The preauricular sinus abscess and periorbital swelling subsided after drainage (Figure 3).

Pus for AFB was positive while culture and sensitivity did not grow any organism. Other tuberculosis (TB) work-ups were negative. On further questioning, the patient denied having prior TB contact. She was then started on a six-month course of anti-tuberculous drugs; two months of an intensive phase with ethambutol, isoniazid, rifampicin, and pyrazinamide, and four months of a maintenance phase with rifampicin and isoniazid. Postoperative review showed a well-healed incision wound. An excision of the preauricular sinus was performed after completion of the anti-tuberculous medication.

Case 2

**Figure 4:**
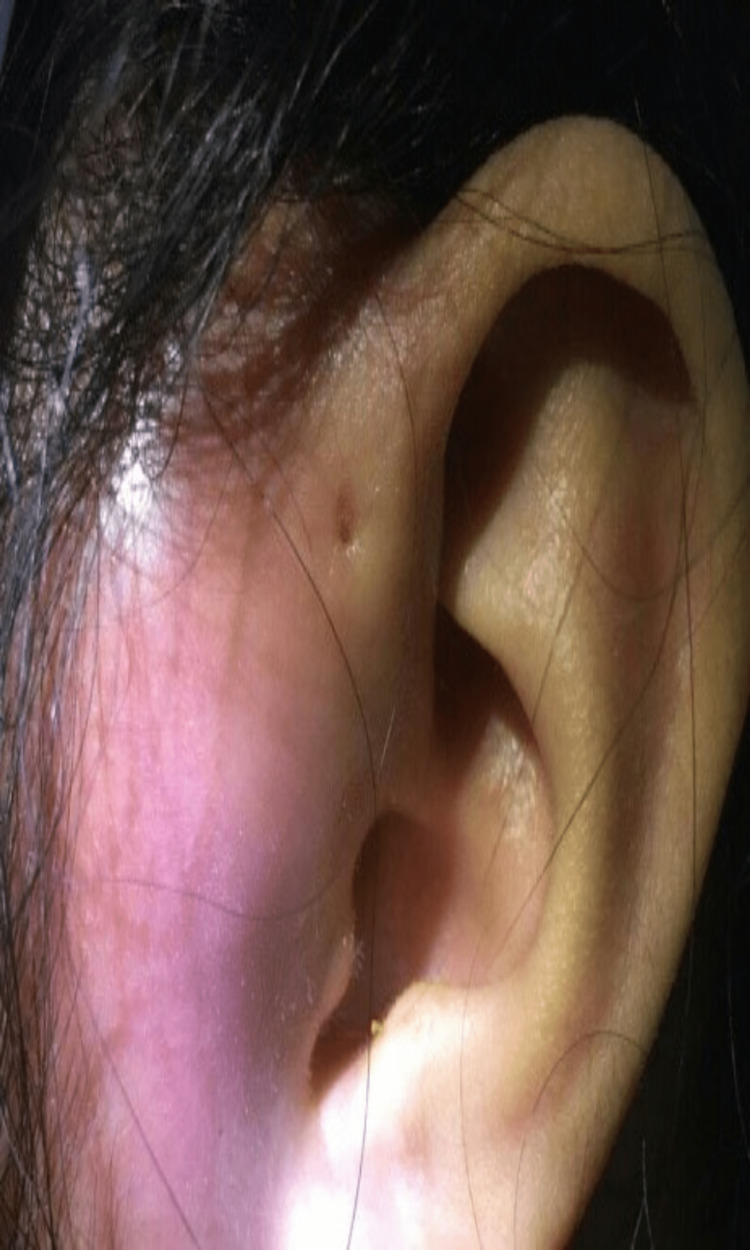
Acute left preauricular sinus swelling.

An eight-year-old boy presented with monthly episodes of recurrent left preauricular abscesses for five months. There was no active discharge from the preauricular sinus opening (Figure 4).

He underwent several incisions and drainage during previous admissions and was treated with oral amoxicillin and clavulanate. The routine bacteriological culture showed no growth. However, the Ziehl-Neelsen (Z-N) staining for AFB smear showed AFB. AFB cultures showed no growth. Other TB work-ups were negative. There was no significant history of TB contact. Subsequently, a six-month anti-tuberculous therapy was initiated; two months of an intensive phase with ethambutol, isoniazid, rifampicin, and pyrazinamide, and four months of a maintenance phase with rifampicin and isoniazid. However, upon completion of treatment, he presented again with another episode of left preauricular sinus abscess. He was subjected to another incision and drainage with the administration of oral cefuroxime axetil. Unfortunately, no sample was sent for TB culture. An excision of the left preauricular sinus was performed after the acute infection had subsided. Histopathological examination of the excised sinus showed no granuloma. After a year of follow-up, there were no signs of recurrence.

Case 3

**Figure 5:**
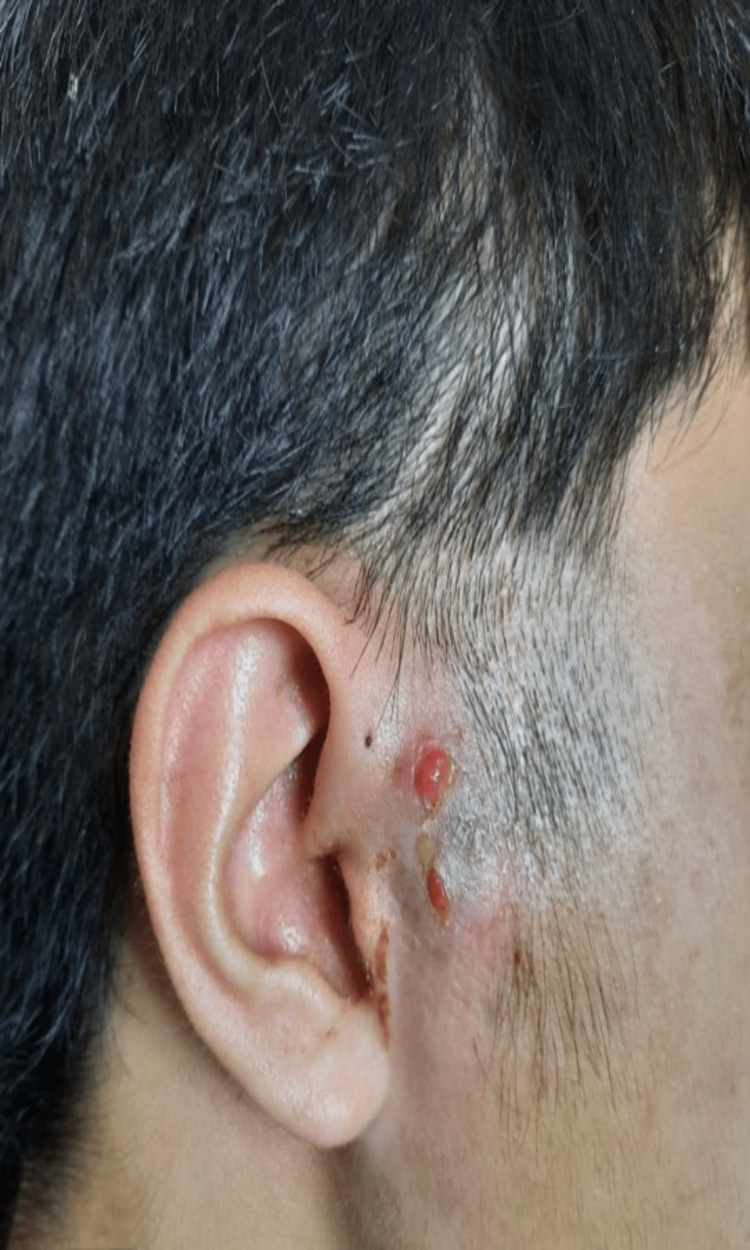
Granulation tissue over the preauricular wound.

A 14-year-old teenager who had multiple right preauricular sinus abscesses presented with a recurrence. Otherwise, he had no active ear symptoms, fever, or history of TB contact. Examination revealed a tender, fluctuant right preauricular swelling measuring 3 x 5 cm. An incision and drainage were performed, which drained 5 ml of pus. The AFB smear culture was positive. His chest X-ray and Mantoux test were otherwise negative. A standard course of anti-tuberculous therapy for extrapulmonary TB was started; two months of an intensive phase with ethambutol, isoniazid, rifampicin, and pyrazinamide, and four months of a maintenance phase with rifampicin and isoniazid. During a postoperative review, there was granulation tissue over the incision scar (Figure 5).

He is electively planned for excision of the right preauricular sinus after completion of anti-tuberculous therapy.

## Discussion

In the general population, the preauricular sinus has an estimated incidence of 0.1-0.9% [1,5,6]. It is commonly seen in patients of Asian and African descent [1,5,7]. Aural TB is less common nowadays. TB of the external ear, especially the preauricular sinus, is extremely rare [5]. Only 10% of TB are extrapulmonary. In the head and neck region, TB is normally observed in cervical lymph nodes and larynx, followed by the oropharynx, salivary gland, and, in a few cases, in the paranasal sinuses, ear, and skin [5,8].

All our patients presented with redness, swelling, and discharge from the preauricular sinus and had no evidence of parotid swelling. However, recurrent episodes of painful persistent swelling prompt patients to seek medical attention [3,9]. When a patient comes in an acute phase of infection, a suitable antibiotic should be administered against the causative organism, and when complicated with a preauricular abscess, patients should be subjected to incision and drainage [3]. In all our patients, no preauricular lymph nodes were identified during incision and drainage.

Our cases were young patients without any immunosuppressive diseases like HIV, diabetes, and autoimmune diseases. Two of them presented with multiple recurrent infections that did not respond to the usual antibiotics. Another case had an unusual presentation of extensive preseptal cellulitis secondary to a preauricular sinus abscess. A sample for AFB should be submitted in patients presented with a refractory preauricular sinus infection or extensive infection, especially in an area where TB is endemic. AFB smears produce a faster detection in comparison to culture and sensitivity [5]. An unresolved infection despite being on a standard antibiotic should raise suspicion of the possibility of an atypical infection like TB.

All our patients were treated empirically with first-line anti-tuberculous therapy as pulmonary and extrapulmonary TB is very rampant in our country even though the *Mycobacterium* pus culture and sensitivity came back as negative. Other TB work-ups were also negative, and we have seen this pattern of infection quite common in our practice. Anti-tuberculous therapy should be commenced according to the recommended regime. In cases of pulmonary TB, treatment consists of two months of intensive phase with ethambutol, isoniazid, rifampicin, and pyrazinamide, followed by four months of maintenance phase with isoniazid and rifampicin [10]. The recommended treatment for extrapulmonary TB is similar to pulmonary TB, which is two months of intensive phase with ethambutol, isoniazid, rifampicin, and pyrazinamide, and four months of maintenance phase with rifampicin and isoniazid for a total of six months in both pediatric patients and adults [10]. The maintenance phase might be longer ranging from four to 10 months if patients have TB meningitis and bone or joint TB [10].

Prompt treatment of empirical anti-tuberculous therapy should be considered once AFB is detected. Liver function should be monitored closely during treatment as anti-TB drugs can cause drug-induced liver injury [10]. For tuberculous preauricular sinus infection, an excision is mandatory during the period of quiescence [11]. One of our cases of recurrent preauricular sinus abscesses, which required multiple incisions and drainage, healed with granulation tissue.

Surgical drainage may result in poor wound healing and it increases the risk of recurrent preauricular swelling despite clearance of AFB in the smear or tissue. Therefore, tuberculous preauricular sinus infection should be treated with anti-tuberculous therapy in combination with an excision.

## Conclusions

A tuberculous preauricular sinus infection should be suspected in patients with recurrent infections that do not respond to standard antibiotics. The AFB smear provides earlier detection of AFB allowing for an empirical anti-tuberculous therapy. To prevent recurrence, a six-month course of anti-tuberculous therapy is recommended, followed by excision of the preauricular sinus once treatment is completed.
